# Enhanced spin Hall effect of tunneling light in hyperbolic metamaterial waveguide

**DOI:** 10.1038/srep30762

**Published:** 2016-08-01

**Authors:** Tingting Tang, Chaoyang Li, Li Luo

**Affiliations:** 1 Information Materials and Device Applications Key Laboratory of Sichuan Provincial Universities, Chengdu University of Information Technology, Chengdu 610225, China; 2 Solorein Technology Inc, Chengdu 610209, China

## Abstract

Giant enhancement of spin Hall effect of tunneling light (SHETL) is theoretically proposed in a frustrated total internal reflection (FTIR) structure with hyperbolic metamaterial (HMM). We calculate the transverse shift of right-circularly polarized light in a SiO_2_-air-HMM-air-SiO_2_ waveguide and analyze the physical mechanism of the enhanced SHETL. The HMM anisotropy can greatly increase the transverse shift of polarized light even though HMM loss might reduce it. Compared with transverse shift of transmitted light through a single HMM slab with ZnAlO/ZnO multilayer, the maximum transverse shift of tunneling light through a FTIR structure with identical HMM can be significantly enlarged by more than three times which reaches −38 μm without any amplification method.

Spin Hall effect of light (SHEL) is the transverse splitting of left- and right-circular components when a linearly polarized light is incident on an interface^1^. The physical mechanism of this phenomenon is the spin-orbit interaction based on the angular momentum conservation law^2^. SHEL is sometimes referred to Imbert-Fedorov (IF) effect as it was theoretically predicted by Fedorov and experimentally confirmed by Imbert^3^,^4^. Due to its potential applications in quantum information and precision metrology, SHEL has recently drawn significant attention of researchers. Theoretical and experimental studies have been carried out in the past decades including various materials and structures^5^,^6^. Generally the transverse shift of transmitted light is on the subwavelength scale and difficult to be directly measured with conventional experimental methods. A transverse shift of transmitted light can be theoretically increased to about 7 wavelengths in a thin epsilon-near-zero metamaterial slab^7^. SHEL in photon tunneling is first proposed by Luo^8^ and is then observed via weak measurements in a three-layer barrier structure^9^ which brings a possibility of spin-based nano-photonic applications. Until now spin Hall effect of tunneling light (SHETL) in a frustrated total internal reflection (FTIR) structure has not been considered in which photonic tunneling may bring large transverse shifts of transmitted light without any amplification method.

Hyperbolic metamaterial (HMM) is a new kind of anisotropic metamaterial formed by stacks of alternating, subwavelength-thin metallic and dielectric layers which can be regarded as an effective uniaxial crystal^10^,^11^,^12^. HMM based on semiconductors of InGaAs/AlInAs superlattice was first demonstrated in 2007^13^. Then a ZnAlO/ZnO multilayer structure was proposed in 2012 which realize negative refractive index in the near-infrared spectral range at about 1.9 μm^14^. Recently Sascha *et al*. demonstrated HMMs operating at telecommunication wavelengths using heavily doped ZnGaO as plasmonic component^15^. HMMs have attracted much attention because they can realize broadband enhancement of spontaneous emission^16^ and imaging below the diffraction limit^17^.

In this paper, we construct a photon tunneling structure of FTIR with HMM and study the SHETL in SiO_2_-air-HMM-air-SiO_2_ waveguide. Theoretical analysis is given based on simulation results including the influences of HMM thickness, loss and dispersion on the transverse shift of circularly polarized light. We show the anisotropy of HMM brings enhanced SHETL and provides an effective method to modulate the splitting between left- and right-circularly polarized light.

## Theory

**Figure 1:**
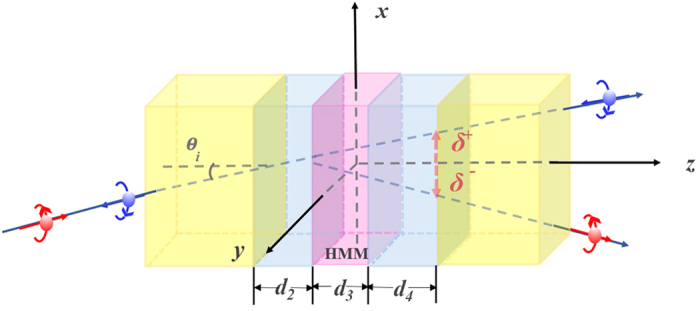
Schematic of SHE of tunneling light in a FTIR structure of SiO_2_-air-HMM-air-SiO_2_.

The photonic tunneling waveguide of FTIR structure composed of a symmetric SiO_2_-air-HMM-air-SiO_2_ waveguide is shown in Fig. 1. Here we assume the incident light is injected into the waveguide in *y*-*z* plane with an incident angle of *θ*. The relative permittivity, permeability and thickness of the media in region 1–5 are denoted by *ε*_*i*_, *μ*_*i*_ and *d*_*i*_ (*i* = 1, 2, 3, 4, 5), respectively in which *ε*_1_ = *ε*_5_ and *ε*_2_ = *ε*_4_. HMM is anisotropic with a relative permittivity tensor of


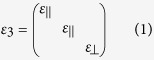


and a relative permeability tensor of


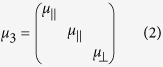


The symmetric waveguide construction satisfies the tunneling condition^18^ of 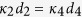, 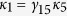 and 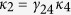 in which 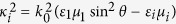, 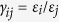 (s-polarized wave) and *γ*_*ij*_ = *μ*_*i*_/*μ*_*j*_ (p-polarized wave). The actual transmission coefficient can be obtained by transfer matrix method. In the same layer, the electric and magnetic fields at any two positions of *z* and *z* + Δ*z* can be related to each other by a transfer matrix^19^ of


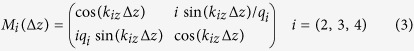


where 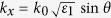 and *k*_0_ is the wave vector in vacuum. For s-polarized wave, 
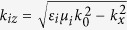
, 
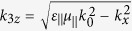
, 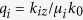 and 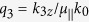 (*i* = 2, 4). For p-polarized wave, 
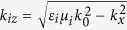
 (*i* = 2, 4), 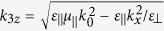, 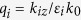 (*i* = 2, 4) and *q*_3_ = *k*_3*z*_/*ε*_||_*k*_0_.

Then, the transmission coefficient *t* can be obtained from the transfer matrix:


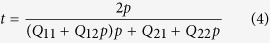


where 
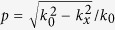
 and *Q*_*ij*_ (*i, j* = 1, 2) is the matrix elements of (*M*_2_*M*_3_*M*_4_). The power transmittance is determined by *T* = tt*.

In our paper, we mainly discuss the spin Hall effect of tunneling mode. The complete tunneling occurs when *κ*_2_*d*_2_ = *κ*_4_*d*_4_ in which 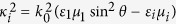 (*i* = 2, 4). Based on the above transfer matrix method, the actual transmission amplitudes in this case can be written as^18^


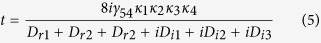


in which


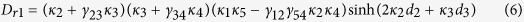



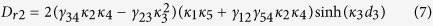



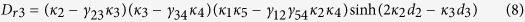



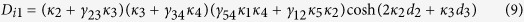



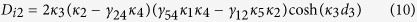



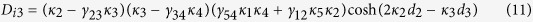


here 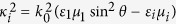 (*i* = 1, 2, 4, 5), 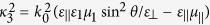, 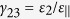 (TM), 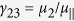 (TE), 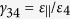 (TM), 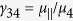 (TE). 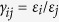 (TM) and 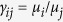 (TE) where *i* = 1, 2, 4 and *j* = 2, 5.

In our paper, the HMM is uniaxially anisotropic in which *ε*_*x*_ = *ε*_*y*_ = *ε*_||_ and *ε*_*z*_ = *ε*_⊥_. As permittivity components in *x*-axis and *y*-axis are identical, the propagation property in HMM can be regarded as the same in isotropic material. In the derivation course of transmitted angular spectrum, transformations of coordinates are used to obtain the relationship between incident field and transmitted field^20^. We can find only two-dimensional rotation matrices are taken into account as the electric field of *z*-axis can be obtained from the divergence equation of *E*_*z*_*k*_*z*_ = −(*E*_*x*_*k*_*x*_ + *E*_*y*_*k*_*y*_). Since the relative permittivity components of HMM in *x*-*y* plane are similar, the transmitted angular spectrum can be regarded as the same as that in multilayered waveguide with isotropic materials^21^.

In our FTIR structure, only HMM in the third layer is uniaxially anisotropic. In our calculation we make use of effective index method to derive the transmitted coefficients of FTIR structure in which 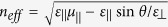 for p-polarized wave. In this case, the influence of HMM anisotropy is included in the calculation of transmitted coefficients. In the deduction course of transverse shift, we find the permittivity difference between *x*(*y*)-direction and *z*-direction only has effect on the transmitted coefficients. Therefore the transverse shifts of left- and right- polarized lights can also be calculated by the method in previous work^2^,^8^,^9^.

In addition, the anisotropic HMM is put in the middle of the five-layer waveguide in our FTIR structure. When light is passing through the HMM layer, the anisotropy of permittivity and permeability brings a different distribution of wave vector in *x, y* and *z* directions as 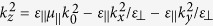. It can be regarded as a modification of spin-dependent splitting in the momentum space induced by HMM waveguide.

In order to obtain an exact analytical result, we give the results without neglecting any terms of electric field in the following analysis. We consider an incident Gaussion beam with angular spectrum of


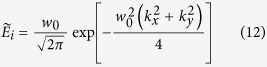


in which *w*_0_ is the beam waist. In our configuration as shown in Fig. 1, the incident plane is *y*-*z* and the transverse shift is along *x*-axis. Thus the angular spectra between transmitted and incident light beams can be written as


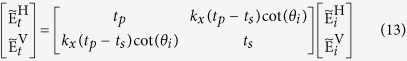


We can get the circular components of the transmitted filed as


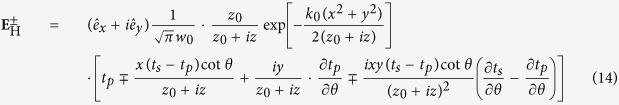


and


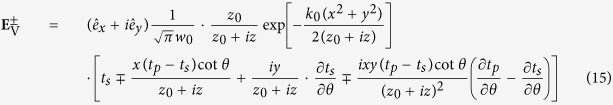


in which 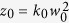 is the Rayleigh length. The transverse shifts of transmitted light can be defined as^7^


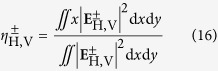


in which *η*^+^ and *η*^−^ indicate the transverse shift of left- and right-circularly polarized components, respectively. We can find the transverse shifts include *z*-dependent and *z*-independent terms which represent special and angular transverse shift, respectively. In our paper, we mainly focus on the spatial transverse shift of the tunneling light. By substituting Eqs (14) and (15) into Eq. (16) (without *z*-dependent terms), the spatial transverse shifts of our FTIR structure can be obtained as


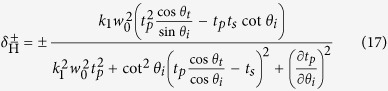



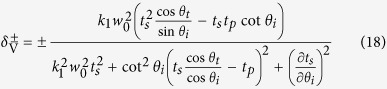


in which *k*_1_ = *n*_1_*k*_0_. Here we notice that when the transmission coefficients are plural, the corresponding transverse shifts should be the real parts of 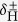 and 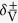. We also notice that if the Fresnel coefficients are plural in our configuration, the transverse shifts should be taken as the real parts of Eqs (17) and (18). Furthermore when 
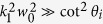
 and 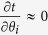, Eqs (17) and (18) can be reduced to the expressions for transverse shifts as


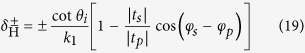



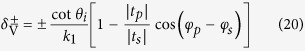


which are similar to that in ref. 9.

As is known to us a linearly polarized beam can be considered as a superposition of right- and left- circularly polarized beams. After transmitted through the FTIR structure, a right (left)-circularly polarized light will convert partially to left (right) one as the transmission coefficients for p- and s- polarized waves are different^7^. Therefore the converted beam component undergoes a shift along *x* axis to ensure the conservation law of angular momentum. The difference between *t*_*p*_ and *t*_*s*_ is proportional to the transverse shift of right- and left- circularly polarized beams as predicted in Eqs (19) and (20). It is easy to see the transverse shift of horizontal (vertical) polarized light is proportional to |*t*_*s*_|/|*t*_*p*_| (|*t*_*p*_|/|*t*_*s*_|). We can also find if the transmission coefficients for p- and s- polarized waves are equal, there will be no transverse shift. A large |*t*_*s*_|/|*t*_*p*_| (|*t*_*p*_|/|*t*_*s*_|) means more right (left)-circularly polarized light is converted into left (right)-circularly polarized light. Thus the circularly polarized light will show a larger transverse shift to conserve the total angular momentum.

## Results

In this section, we give some simulation results to discuss the enhancement of SHETL in the FTIR structure. In order to obtain an exact analytical result, the transverse shifts of circularly polarized light in this paper are calculated without neglecting any terms.

In the SiO_2_-air-HMM-air-SiO_2_ waveguide, we first ignore the dispersion of HMM and assume the relatively permittivity is positive. The silica is weakly dispersive with a dispersion relation of


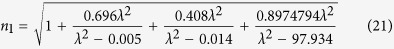


**Figure 2:**
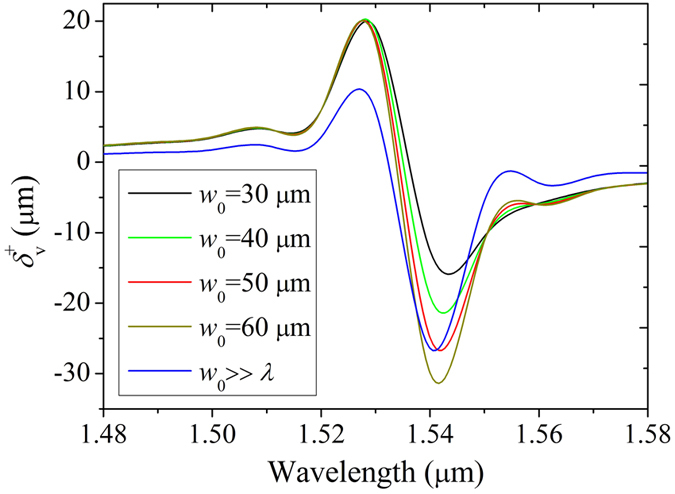
Transverse shift spectrum of vertical polarization input for different beam waists. The parameters of FTIR structure are chosen as *ε*_2_ = 1, *ε*_||_ = 1.5, *ε*_⊥_ = 2, *θ* = 61° and *d*_2_ = *d*_3_ = 1 μm. The blue curve shows the transverse shift for *w*_0_ ≫ *λ.* In this case its positive peak is smaller than that of 30 μm ≤ *w*_0_ ≤ 60 μm while its negative peak is close to that of *w*_0_ = 50 μm.

In this paper, all the materials are non-magnetic which mean *μ*_1_ = *μ*_2_ = *μ*_3_ = 1. We choose *ε*_2_ = 1, *ε*_||_ = 1.5, *ε*_⊥_ = 2, *θ* = 61° and *d*_2_ = *d*_3_ = 1 μm. We first take into account the influence of beam waist *w*_0_ on the transverse shift as shown in Fig. 2. When *w*_0_ equals 30 μm, 40 μm, 50 μm and 60 μm, the transverse shifts of vertical polarization can be calculated by Eq. (18) without neglecting any terms. But when *w*_0_ is much larger than the incident wavelength, Eq. (20) can be used to calculate the transverse shift approximately as the blue curve in Fig. 2. It is easy to see with the increase of beam waist, the negative transverse shift peaks are enlarged from −12.55 μm to −29.90 μm which appear at the same wavelength of 1.54 μm. While when *w*_0_ is much larger than *λ*, the negative transverse shift peak is reduced to 26.39 μm which is close to that for *w*_0_ = 50 μm. Therefore for simplicity and precision, we choose the beam waist as 50 μm in the following simulations and calculate the transverse shifts with no approximation in order to obtain convincing results.

**Figure 3:**
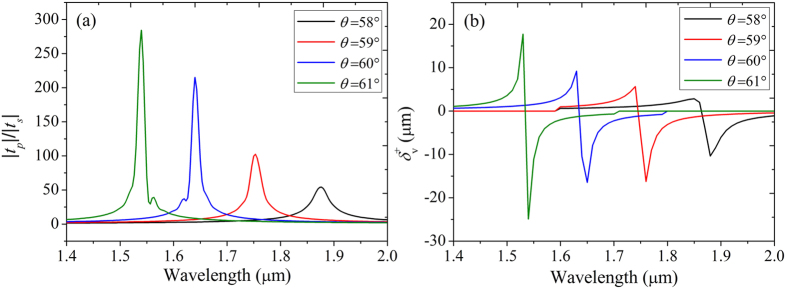
Transmittance ratio (|*t*_*p*_|/|*t*_*s*_|) and transverse shift spectrum of vertical polarization input for different incident angles. The parameters of FTIR structure are chosen as *ε*_2_ = 1, *ε*_||_ = 1.5, *ε*_⊥_ = 2 and *d*_2_ = *d*_3_ = 1 μm. There is a one-to-one correspondence between a transmittance ratio peak and transverse shift peak.

Then we give the transmittance ratio of |*t*_*p*_|/|*t*_*s*_| of tunneling modes for different incident angle in Fig. 3(a). In a small incident angle range, the increase of *θ* enlarges the transmittance ratio of tunneling mode. The corresponding transverse shifts of vertical polarization are shown in Fig. 3(b). In this case, the transmittance of s-polarized component is close to zero and most of the light is reflected back. We can find the transverse shift peak appears at the wavelength with a transmittance ratio peak of |*t*_*p*_|/|*t*_*s*_|. In a wavelength range from 1 μm to 2 μm there is only one transverse shift peak for a special incident angle. Meanwhile there is a one-to-one correspondence between a transmittance ratio peak and transverse shift peak. In addition, with the increase of the transmittance ratio peak, the transverse shift also increases. This phenomenon verifies the prediction in section 2 that the SHETL is associated with the transmitted coefficients of both horizontal and vertical polarizations.

**Figure 4:**
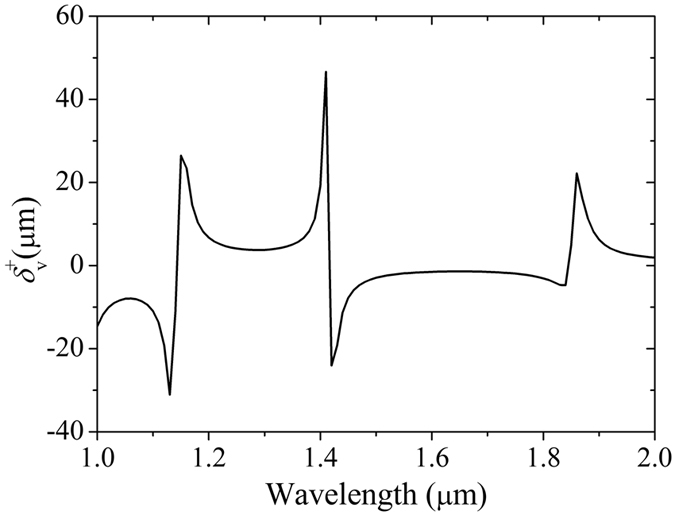
Transverse shift spectrum of vertical polarization input for different wavelengths. The parameters of FTIR structure are chosen as *ε*_2_ = 1, *ε*_||_ = 1, *ε*_⊥_ = −1, *d*_2_ = 1 μm and *d*_3_ = 2 μm. More tunneling modes are generated in anisotropic waveguide with negative component compared with positive permittivity.

Then we study the transverse shift in the FTIR structure when the vertical component of permittivity in HMM is negative. Here we choose *ε*_2_ = 1, *ε*_||_ = 1, *ε*_⊥_ = −1, *θ* = 49°, *d*_2_ = 1 μm and *d*_3_ = 2 μm. The transverse shift spectrum of vertical polarization input for different wavelength is shown in Fig. 4. We can find when *ε*_⊥_ is negative three tunneling modes appear in the wavelength range from 1 μm to 2 μm. This phenomenon is induced by the negative component of HMM permittivity. It can be speculated that more tunneling modes are generated in anisotropic waveguide with negative component compared with that with only positive permittivity components.

**Figure 5:**
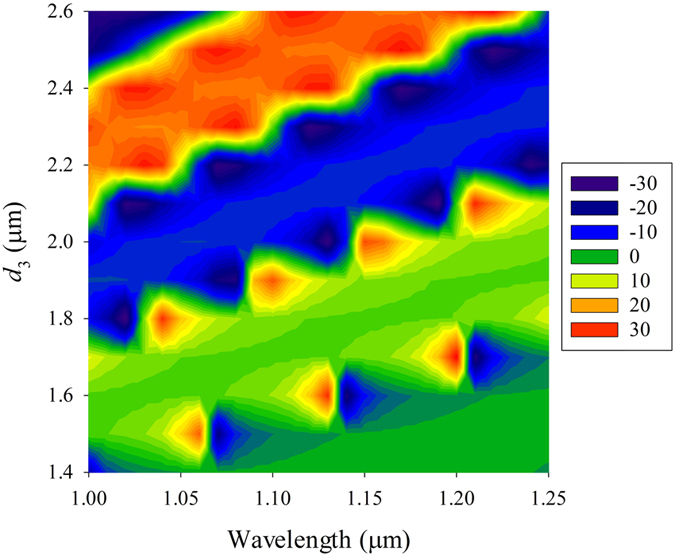
The transverse shift contours of the FTIR structure for vertical polarization input when HMM thickness is different. The parameters of FTIR structure are chosen as*ε*_2_ = 1, *ε*_||_ = 1, *ε*_⊥_ = −1 and *d*_2_ = 1 μm. Periodic transverse shift groups and band can be observed in this figure.

In the following we explore the influence of HMM thickness on SHETL. Here we choose *ε*_2_ = 1, *ε*_||_ = 1, *ε*_⊥_ = −1, *θ* = 49° and *d*_2_ = 1 μm. The transverse shift contour of vertical polarization for different *d*_3_ is shown in Fig. 5. When the HMM thickness is between 1.4 μm and 1.6 μm, there are three transverse shift peaks appear at *λ* = 1.06 μm, 1.14 μm and 1.21 μm, respectively. These peaks have shifts to larger incident wavelength with the increase of HMM thickness. When 1.7 μm < *d*_3_ < 2.1μm, another group of transverse shift peaks appears which includes four obvious peaks in the same wavelength range from 1.0 μm to 2.0 μm. With the increase of HMM thickness there is a large transverse shift band in the transverse shift contour. We can observe large transverse shifts within a range of wavelength for a special incident angle and HMM thickness. For example, when *d*_3_ = 2.4 μm a large transverse shift of right-circularly polarized light can be obtained for an incident wavelength range of 1.00 μm to 1.15 μm. The transverse shift band may be induced by the convergence of two groups of periodic transverse shift peaks. This unique phenomenon has not been observed in other isotropic waveguides. The periodic transverse shift peaks may have potential applications in band pass filters.

Further calculation results show that the thickness change of air layer has no effect on the transverse shift. This brings us a large flexibility of design for the FTIR structure which shows a good tolerance for dimension error in fabrication process.

**Figure 6:**
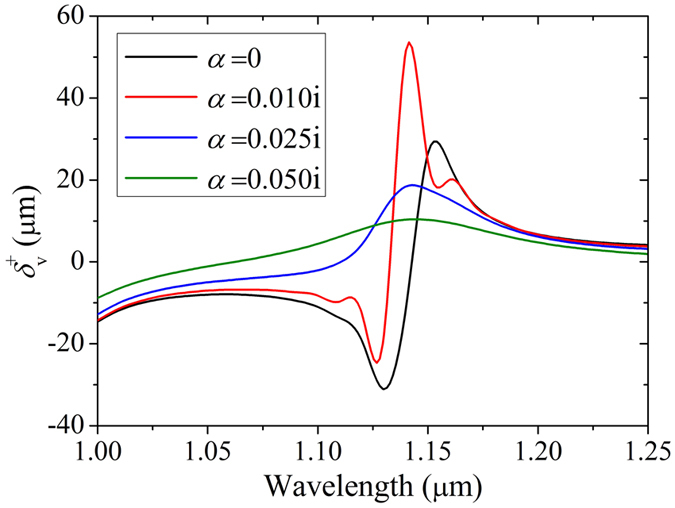
Transverse shift spectrum of vertical polarization input for different attenuation coefficients. The parameters are chosen as *ε*_2_ = 1, *ε*_||_ = 1 + *α, ε*_⊥_ = −1 + *α, d*_2_ = 1 μm and *d*_3_ = 2 μm. A slightly lossy HMM brings an enhancement of SHETL while in a heavily lossy HMM waveguide no obvious transverse shift can be observed as the absorption of incident light.

As HMM is always lossy, we should study the influence of absorption of metamaterial on SHETL. Here we assume both attenuation coefficients of parallel and vertical HMM permittivity equal to *α* which means *ε*_||_ = 1 + *α* and *ε*_⊥_ = −1 + *α*. We also choose *ε*_1_ = 2.085, *ε*_2_ = 1, *θ* = 49°, *d*_2_ = 1 μm and *d*_3_ = 2 μm. When *α* changes from 0 to 0.05i, the transverse shift for vertical polarization is shown in Fig. 6. When the HMM is lossless, the transverse shift peaks are about ±30 μm. While when HMM is slightly lossy with an attenuation coefficient of about 0.01i, the positive transverse shift peak is enlarged. Then with the increase of attenuation coefficient the transverse shift peak is reduced. Further calculation shows even an attenuation coefficient of about 0.1i evanishes the transverse shift peak. It is easy to see the HMM loss has a great influence on the transverse shift. We also notice the exact value of transverse shift peak is associated with the transmission coefficient ratio between s- and p- polarized lights. Therefore a slightly lossy HMM brings an enhancement of SHETL while in a heavily lossy HMM waveguide no transverse shift can be observed as the absorption of incident light.

At last, the material dispersion is taken into account as HMM is strong dispersive. In order to make our results convincing, we choose a recently reported HMM operating at telecommunication wavelengths using heavily doped ZnGaO as plasmonic component^15^. The dispersion of anisotropic metamaterial can be described as^15^


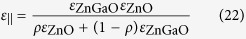



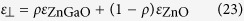


where *ε*_ZnO_ = 3.7 and *ρ* = *d*_ZnGaO_(*d*_ZnGaO_ + *d*_ZnO_)^−1^. Here the permittivity of ZnGaO is described by Drude’s dielectric function


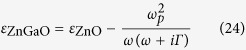


in which *ω*_*p*_ is the plasma frequency and *Γ* is electronic damping rate. According to Ref. 15, other parameters are chosen as *ħω*_*p*_ = 1.88 eV, *Γ* = 112 meV and *d*_2_ = 1.7 μm.

**Figure 7:**
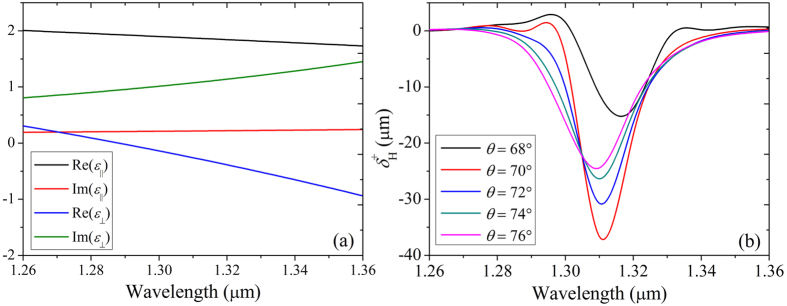
(**a**) The dispersion curves of HMM and (**b**) transverse shifts of right circular component of transmitted light for H-polarization input in FTIR structure. A large transverse shift peak can still be available even the strong absorption occurs in the waveguide.

The dispersion curves of HMM is shown in Fig. 7(a) when the wavelength is between 1.26 μm to 1.36 μm. We also give the corresponding transvers shift spectrum in Fig. 7(b) in which a maximum transverse shift of about −38 μm appears at *λ* = 1.31 μm and *θ* = 70°. In this case, the permittivity components are *ε*_||_ = 1.8713 + 0.2163*i* and *ε*_⊥_ = −0.2598 + 1.0798*i*. The attenuation coefficient of HMM is obviously much greater than that we discussed in Fig. 6 which is less than 0.1i. However, the reduction of transverse shift peak can be compensated by the anisotropy of HMM. It also verifies the prediction that the transverse shift is associated with the transmitted coefficients of both p- and s- polarized light. Once a large value of |*t*_*s*_|/|*t*_*p*_|(|*t*_*p*_|/|*t*_*s*_|) can be realized, a large transverse shift peak can be observed. Therefore, a large transverse shift peak is still available even strong absorption occurs in the waveguide. In addition, compared with transverse shift of transmitted light through a single HMM slab, the transverse shift of tunneling light through a FTIR structure is significantly enlarged. Further calculation shows the maximum transverse shift of transmitted light through a slab with the same HMM of ZnAlO/ZnO multilayer is smaller than 10 μm. By use of FTIR structure the maximum transverse shift of tunneling light can be enlarged by more than three times. Therefore we can get a conclusion that SHETL in a FTIR structure can be greatly enhanced by HMM even a large absorption occurs.

## Conclusion

In this paper, we theoretically study the enhancement of SHETL in a FTIR structure with HMM. The transverse shift of right-handed polarized light in a SiO_2_-air-HMM-air-SiO_2_ waveguide is calculated and the physical mechanism of the enhanced SHETL is discussed. The influences of HMM thickness, loss and dispersion on the transverse shift of left- or right-circularly polarized light are also analyzed. Simulation results show HMM anisotropy can significantly increase the transverse shift of tunneling light even though HMM loss might reduce it. Compared with transverse shift of transmitted light through a single HMM slab with ZnAlO/ZnO multilayer, the maximum transverse shift of tunneling light through a FTIR structure with identical HMM can be significantly enlarged by more than three times which reaches −38 μm without any amplification method. The FTIR structure provides an effective method to enhance the SHEL which also facilitate the potential applications in nano-photonic devices.

## Additional Information

**How to cite this article**: Tang, T. *et al*. Enhanced spin Hall effect of tunneling light in hyperbolic metamaterial waveguide. *Sci. Rep.* **6**, 30762; doi: 10.1038/srep30762 (2016).
